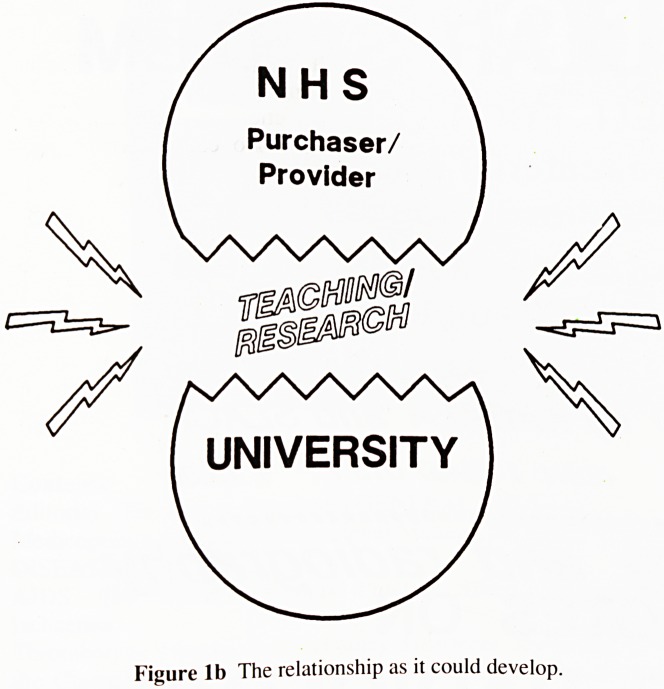# The Changing Face of Bristol Medicine

**Published:** 1991-06

**Authors:** 


### The Changing Face of Bristol Medicine

### A Symposium Organised by the Bristol Medico-Chirurgical Society and held at Southmead Hospital on 13 February 1991, under the Chairmanship of Prof R. Langton Hewer. Most of the speakers have kindly submitted abstracts of their papers as follows.

### GENERAL PRACTICE IN TURMOIL

**Michael Whitfield**
Senior Lecturer (Gen. Pract.) Univ. Bristol.

The last two years have seen major changes occurring in general practice. We have seen the so-called "conservative'' government introducing major, untested changes with the express belief that the changes will improve the quality of care that general practitioners are providing; that the changes will give patients greater and more informed choice and that the rationing of health care is to move from the hospital service to the general practitioner.

We have already seen the arrival of the general manager replacing the administrator, targets replacing the items of service payment, higher capitation fees and the reduction of the basic practice allowance.

We now need to organise practices to be able to send out letters inviting all those over 75 (nearly 1000 in our practice of 5 doctors) to receive a visit from a health worker to the patients home every year, to measure the height and weight and BP of every new registrant to practice (however often he or she changes practice), to offer every patient a health check every 3 years if they haven't been seen and to provide a detailed annual report every year to the general manager.

We have seen the development of the practice manager, nearly 80% of practices in Avon are computerised, Practice nurses are running clinics for diabetics, asthmatics, well people and people under stress.

As if all of this is not enough from April this year we are faced with indicative drug budgets for each practice (which theoretically we should all have been discussing and agreeing with an officer of the FHSA)

For some practices there will be the excitement and challenge of fund-holding and somehow the FHSA general manager will be expected to check that all of the new contract is being honoured by each practice.

Finally during this year each doctor has to be seen to be taking part in medical audit.

Ladies and gentlemen, I hope that I have painted a picture of turmoil. It is certainly an uncomfortable time for general practice. We have this afternoon, three speakers who are going to share with you some of their concerns and hopes for the future.

### GENERAL PRACTICE AND THE NEW CONTRACT — BETTER TO TRAVEL HOPEFULLY?

**Paul and Joy Main**
Hartcliffe Health Centre, Bristol

#### The New Contract — general

Any time of accelerated change, such as we are currently moving through as a profession, is full of creative potential. But for that potential to be realised, meticulous care needs to be paid to all aspects of the management process — identifying the aims; setting the objectives — the "way-markers'' on the path towards the aims; and defining the process through which the aims will be achieved. Pivotal keystones in the process are the creative use of motivational and communication skills. These have been conspicuously absent in government's dealing with the profession, and have left us with a sense that government does not value us. We have to be able to own, and deal with, the resulting anger in order to be free enough to look creatively at the issues raised by the new contract. We have tried to look as objectively as we can at those issues, and would like to share that with you.

*The contract's aims* (never openly communicated to the profession) seem to be:

a) to draw all primary care up to the level of the best;
b) to turn primary care proactive;
c) to increase the importance of the preventive agenda; and
d) cost containment and value for money

We find no quarrel with these aims, but retain the anxiety that the "outerness'' and "measurability'' they embody risk squeezing the space to listen and to care, out of the agenda. "Not everything that can be measured, matters — and not everything that matters, can be measured.''

*The objectives* present more objective problems:

a) New patient checks: the parameters we are contractually required to check have questionable scientific validity.
b) Three-yearly checks offered to non-attenders: there is little evidence that these are other than a waste of time.
c) Annual, in-depth checks on over -75's: these have been shown to yield little redeemable pathology. The need they uncover is often socioeconomic, and resources may well not exist to address it.
d) The target concept for resourcing immunisations and cervical smears risks introducing unacceptable stress into the doctor/patient relationship.

With regard to *the contract's process* —

a) The chosen motivational tool is the movement of financial resourcing. The implications are profound for the ability of G.P.'s, as independent contractors, to resource the care they give.
b) Opening health care up to the forces of the free market means
   i) the fittest practices — and patients — will survive and prosper, and the most vulnerable situations (almost always the most deprived) risk deteriorating standards of care.
   ii) the corollary of being rewarded for attracting more patients to our lists is that we shall be required to do the minimum acceptable for the maximum number. This is not how many of us want to practice.
c) Government's motivational skills are testified to by the desperately low morale within the profession, and drops in number and calibre of recruits.
d) Responsible use of initiative by practitioners and FHSA general managers is being severely curtailed by government.

#### The new contract and deprivation

The concepts of the contract are middle-class — a charter for the advantaged. Social classes IV and V experience vastly more morbidity and premature mortality than average, and the health/wealth gap is widening. The obvious need this brings for greater resourcing of health care to deprivation, if the deprived are to have "an average chance of health'', has never been institutionally recognised by health resource providers, and the struggle to deliver effective health care in deprivation is correspondingly greater. Deprivation is inherited, and socioeconomic and educational deprivation are tightly linked.

Perceptual inability to set medium-term goals means health promotion has to be opportunistic and oft-repeated, but the contract doesn't resource this model of health promotion. A recent large survey in our area showed that while government and the middle classes think smoking, alcohol, diet and exercise are the most important factors bearing on health, the deprived identify housing, unemployment, lack of leisure facilities and environmental pollution as their problems. The government and the deprived have two different agendas.

Government is currently only resourcing preventive care carried out in clinics. Because the deprived can't organise their lives to attend clinics, and they don't share middle class health promotion goals, there is a danger of prevention resources haemorrhaging into middle class areas, where they will have least impact on premature mortality and morbidity, and on the health-wealth divide.

Targets are harder to achieve in deprivation, because the unsmearable and unimmunisable are concentrated among the educationally deprived. And yet higher levels of smears and immunisations are inarguably more important, because of the coexistent higher morbidity.

The "deprivation allowance'' is a new — and welcome — concept of differentially resourcing health care in deprivation. Currently, it is very inaccurately targeted. The data used is ten years out of date, allocated on the basis of electoral wards, whose sizes vary wildly, and its parameters were not originally meant to measure deprivation, at all. Hartcliffe and Withywood, one of the two most deprived areas of Bristol, currently receive no allowance at all.

The new contract, then, needs much modification if its demands are to match real health needs. Particularly with regard to its failure to adequately address the health needs of the deprived, only radical review will enable the nineties to be the decade in which the health-wealth gap started to narrow, and the deprived started the journey towards "an average chance of health''.

### THE PURCHASERS' TASK AND HOW IT WILL BE PERFORMED

**Ann Lloyd**
Dist. Gen. Manager, Frenchay Health Dist.

The NHS Community Care Act heralded the advent of the greatest reform since the establishment of the NHS in 1948, of which the main objective was to give patients better health care and a greater choice of services available. It required a change in emphasis of the role of District Health Authorities away from a concentration on the provision of health care services to the identification of health care requirements and the purchasing of services.

There are four key tasks for purchasing Health Authorities which are:

1. To develop a method of assessing both met and unmet health needs of the population. This requires knowledge of the health of the population together with an understanding of the natural history of disease, the effectiveness and relative priority of health service intervention, knowledge of actual and potential availability of health services and the use of health services by the population.
2. To develop a health care plan in response to these assessments of needs in close liaison with GPs on whose behalf DHAs are purchasing services for their patients. The DHA must understand what service is required by GP's in order to achieve the aim of a sound link between primary and secondary care.
3. To develop a specification for services once the needs of the population and the views of GPs are understood.
4. To develop monitoring systems which can respond effectively to the complex new system covering volume, cost and quality.

The introduction of purchasing' for a population has re-focused attention on the populations' needs for health services on the requirements of GPs.

As purchasing Health Authorities become more explicit, needs which remain unmet and services not provided will be identified and will be the subject of debate inside and outside the NHS.

### CARING FOR PATIENTS — A CLINICIAN'S VIEW

**James D. Wisheart**
Con. Cardiac Surgeon, Bristol, R.I.

As a clinician, I shall present a pragmatic view of the NHS Act, asking the question, "In what way will we do our work within the new structures?'' In as much as the future is uncharted, my view will be speculative, and also we should be mindful that at a time of radical change there is much which will remain the same, and this continuity may be caricatured by the conviction that in the years to come we will still be trying to extract a quart from a pint pot.

The focal point of change is the internal market — purchasers and providers, contracts and audit. This will affect ALL clinicians whether their provider units are managed directly or by an NHS Trust. The contracts will specify an agreed volume of work, for an agreed sum of money, subject to audit. There is concern about patients "beyond the Contract'' — that is patients who need the service, but do not obtain it because the total need in the Community is greater than the volume of service bought by the purchasers for that Community. *Who* will decide which patients to treat and which not to treat? *Who* will keep a record of patients not treated, so that our total health care need may be estimated? *What* is the responsibility of provider clinicians for patients beyond the contract — do they have any?

Secondly, the Clinician has an interest in directorates — that small functional unit which makes and fulfils contracts, usually having a doctor as director. I believe that the combination of a small "grass-roots'' unit with which its members can all readily identify, together with some autonomy over how the income from its contract should be used, has the potential to release great creativity. This potential creativity stems from the idea of autonomy. I believe that there will always be tension between having more or having less autonomy, and that we as clinicians should press for more. The price of less autonomy, will be demotivation, the loss of potential creativity, and therefore the loss of increased efficiency and competitiveness.

We clinicians must be pragmatic, seeking to make the new arrangements work, in order to care for our patients.

### A BETTER HEALTH SERVICE? — A UNIVERSITY VIEW

**Gordon M. Stirratt**
Prof of Obstetrics and Gynaecology, Dean of the Faculty of Medicine, Univ. of Bristol

The current NHS reforms are the most sweeping since its inception in 1948. Their main features have been described by previous contributors. They are so cataclysmic that no part of the Health Service is spared and far too little thought was given to their knock-on effects before they were promulgated. This is particularly so for those aspects which affect the teaching and training of medical students and junior staff. This article considers the reforms from a university view point. The views expressed are personal and do not necessarily reflect the policy of the University of Bristol.

### Medical student teaching

For over two hundred years we have relied on the apprenticeship system for our clinical teaching. It has been modified considerably over the years but the principles remain the same — the student is part of the team involved in patient care and he or she learns as the consultant team manages the patients under their care. Particularly in the early stages of the clinical curriculum, when the young student is being introduced to such basic skills as history-taking and the fundamental principles of Surgery and Medicine, this system depends on a balanced case-mix and time for the student to be with the patient at all stages of his or her illness. Over the past ten to fifteen years the pattern of patient care has changed out of all recognition and mostly for the better. Much more care occurs in "the community'' (but we must not forget that the hospital is part of the same community) and patients are in hospital for much shorter periods than ever before. The result is that the medical student in the hospitals used for teaching is now seeing an unbalanced range of conditions and is unable to follow the same patient through from presentation to discharge. In addition the financial consequences of the low priority status of acute services has led to the recurrent closure of surgical or medical beds often at crucial times for student teaching. As their teachers we are concerned that their experience will be incomplete for future practice and that they will not adequately learn how the patient is to be cared for in the hospital setting.

The purchaser-provider split (especially budget holding GPs) could greatly exacerbate an already difficult position for student teaching without positive direction from the Department of Health (DOH) and the Regional Health Authority. The latter is now required to ensure that appropriate facilities are provided for teaching and research. There are, however, measures which can help to alleviate the problem given the finances to implement, organise and monitor them. For example, more teaching could take place in some regional District General Hospitals. We must strengthen our teaching links with General Practice. The University is already committed to develop this as an academic speciality despite the centrally imposed financial constraints on universities discussed below. An increased exposure to General Practice is, however, not the sole solution. The case-mix would remain unbalanced and the "density'' of specific conditions is very light. *Each discipline must recognise its role and that of others as a setting for teaching in the overall context of a balanced curriculum.* The Teaching Hospitals will continue to play a key role in teaching and we must, therefore, use the available resources to best advantage. There must also be greater communication between those of us responsible for clinical teaching and clinical directors and hospital managers to, for example, reduce the impact of bed closures.

### The medical curriculum

Concurrent with the NHS reforms there is a realisation that the undergraduate curriculum suffers from severe information overload. It undergoes continual modification but more radical change is necessary and is likely to be required by the General Medical Council. High on the agenda is discussion about the establishment of a "core curriculum'' with the student able to choose from among a whole series of further options.

### Resources for teaching

Major changes in the curriculum will have inevitable effects on the teaching resource. The current demand by the Government for efficiency savings in the universities can only be achieved by taking more students, having fewer teachers or generating income from elsewhere. Since there is an official ceiling on the number of home and EC medical students, the pressure is to reduce the teaching establishment. Bristol already has one of the smallest teaching establishments among Medical Schools and we must, therefore, concentrate on income generation rather than staff reduction. There is, regrettably, a further complication. The Universities' Funding Council (UFC) uses a complex formula to determine the grant to the University. This is, in part, determined by an assessment of each department's research performance on the basis of grants awarded and publications in peer review journals. This applies equally to the Schools in the Medical Faculty. It is vital for our financial health that our UFC Research Rating improves. Unfortunately, research has no natural place within the purchaser-provider split. Clinical academic staff, therefore, find themselves having to accommodate the conflicting requirements of the UFC and the DOH (with the unfortunate medical student sandwiched in the middle) and with the threat of fewer resources to achieve the required objectives. The figure illustrates the problem we may face. *See p. 55.*

### Dealing with change and uncertainty

"Working for Patients'' may do just that. Even now it seems clear that it is a mixture of the good, the bad and the uncertain. Indeed the major (and surely avoidable) problem was that so much was thrown up into the air at the same time. Any psychologist will affirm that the most effective way of causing stress and anxiety is to produce the greatest amount of uncertainty for the longest possible time. This is exactly what has happened in the Health Service. Radical changes covering the structure and management of the Hospital, Community Care and Primary Health Care services, Nurse Training (Project 2000), Medical Postgraduate and Continuing Education, Junior Staff Training (Achieving a Balance), Junior Hours of work and, in the context of this article, the structure and financing of the Universities were proposed and introduced in short order. Many of these could be beneficial if properly resourced but together they have produced maximum uncertainty and loss of morale. The stress and anxiety among nursing, medical and managerial staff has been plain to see but seemed to go unrecognised.

Now that April 1st has ushered in this Brave New World, let us hope that the changes do indeed produce the promised "Better Health Service'' without sacrificing our future heritage. From past experience, let us also hope that we are not subjected to yet further reorganisation just as this one is beginning to bear fruit!

*Diagrams illustrating relationship between NHS and University are on p. 55.*

### THE CHANGING FACE OF BRISTOL MEDICINE

*Continued from p. 32.*

**Figure 1a** The relationship between NHS and University as it should be.

**Figure 1b** The relationship as it could develop.

### TOWARDS BETTER DISCHARGE SUMMARIES

*King*

West of England Medical Journal Volume 106)ii) June 1991

## Figures and Tables

**Figure 1a f1:**
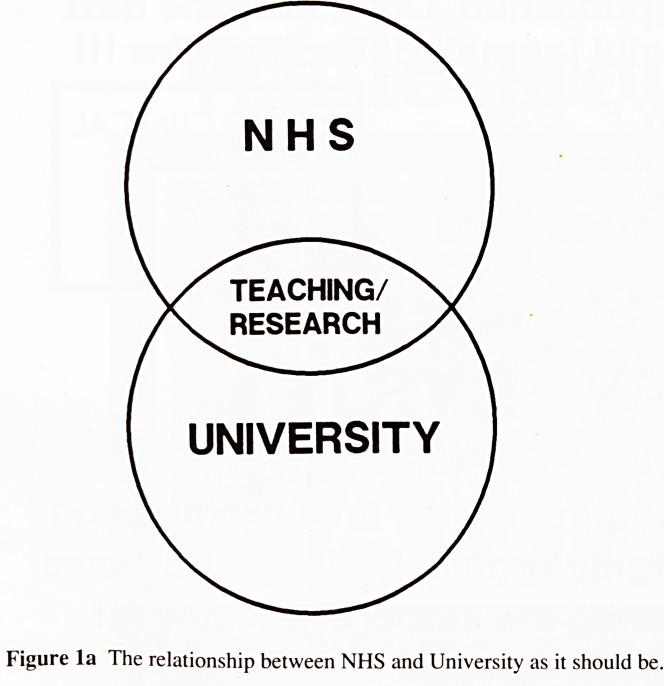


**Figure 1b f2:**